# Kazinol C from *Broussonetia kazinoki* stimulates autophagy via endoplasmic reticulum stress-mediated signaling

**DOI:** 10.1080/19768354.2021.2023628

**Published:** 2022-01-10

**Authors:** Yunkyeong Lee, Junhee Kwon, Ji Hye Jeong, Jae-Ha Ryu, Keun Il Kim

**Affiliations:** a Department of Biological Sciences and Cellular Heterogeneity Research Center, Sookmyung Women’s University, Seoul, Republic of Korea; b Research Institute of Women’s Health, Sookmyung Women’s University, Seoul, Republic of Korea; c College of Pharmacy, Sookmyung Women’s University, Seoul, Republic of Korea

**Keywords:** Kazinol C, endoplasmic reticulum stress, unfolded protein response, autophagy, apoptosis

## Abstract

Autophagy modulators are considered putative therapeutic targets because of the role of autophagy in cancer progression. Kazinol C, a 1,3-diphenylpropane from the plant *Broussonetia kazinoki*, has been shown to induce apoptosis in colon cancer cells through the activation of AMPK at high concentrations. In the present study, we found that Kazinol C induced autophagy through endoplasmic reticulum stress-mediated unfolded protein response signaling in several normal and cancer cell lines at low concentrations of Kazinol C that did not induce apoptosis. Kazinol C activated the transducers of unfolded protein response signaling, leading to target gene expression, LC3-II conversion, and TFEB nuclear translocation. Chemical inhibition of endoplasmic reticulum stress reduced LC3-II conversion. In addition, blockade of autophagy by knockout of *Atg5* or treatment with 3-MA enhanced Kazinol C-induced apoptosis. In summary, we have uncovered Kazinol C as a novel autophagy inducer and confirmed the role of autophagy as a cellular stress protector.

## Introduction

Autophagy is an internal cellular degradation and recycling process in which subcellular compartments are digested by lysosomes and vacuoles when cells need energy in response to stress conditions such as starvation (Doherty and Baehrecke 2018). In addition, autophagy plays a vital role in cellular homeostasis through the lysis of damaged organelles as well as unfolded, misfolded, and aggregated proteins (Dikic and Elazar 2018). Macroautophagy (hereafter referred to as autophagy) is a well-known type of autophagic process, in which a double-membrane structure called autophagosome engulfs target organelles or proteins, and then the autophagosome and lysosome fuse to form an autolysosome (Galluzzi et al. 2017). The contents are broken down by lysosomal enzymes (Galluzzi et al. 2017). Recent studies have shown that dysregulation of the autophagic process is associated with many human disorders, including neurodegenerative diseases, aging, metabolic disorders, immune dysfunction, and cancer (Mizushima and Levine 2020; Klionsky, Petroni, et al. 2021). Therefore, autophagy modulators are being developed as therapeutic agents for various diseases.

The endoplasmic reticulum (ER) is a multifunctional organelle that specifically synthesizes proteins, lipids, and steroid hormones, as well as regulates protein fidelity and calcium ion homeostasis within cells (Schwarz and Blower 2016). Moreover, it facilitates autophagosome formation (Klionsky, Abdel-Aziz, et al. 2021). When various sensors recognize perturbations, such as misfolded/unfolded protein accumulation, downstream signaling cascade called unfolded protein response (UPR) is activated, which relieves ER stress and modulates ER function and homeostasis (Hetz 2012; Smith and Wilkinson 2017). Otherwise, cells die by the activation of apoptotic signaling (Hetz 2012; Smith and Wilkinson 2017). The UPR consists of three signaling pathways, each containing a sensor molecule, namely inositol-requiring enzyme 1α (IRE1α), protein kinase RNA-like ER kinase (PERK), and activating transcription factor (ATF) 6 (Smith and Wilkinson 2017).

It is becoming evident that autophagy can be activated by ER stress signals and can contribute to resolving damaged conditions favorable for cell survival (Smith and Wilkinson 2017). Well-known pharmacological UPR inducers such as tunicamycin, thapsigargin, A23187, and brefeldin A, have been shown to induce autophagosome formation in mammalian cells (Ogata et al. 2006; Ding et al. 2007). We have also previously reported that a lignan [(−)-(2R,3R)-1,4-O-diferuloylsecoisolariciresinol, DFS] from *Alnus japonica* induced UPR and ER stress signaling, resulting in the activation of the autophagic process (Kwon et al. 2020). In addition, inhibition of autophagy sensitized DFS-induced ER stress-mediated cell death (Kwon et al. 2020). Numerous studies have shown that targeting autophagy triggered by natural compounds is an excellent strategy for the treatment of many diseases, including cancer (Bae et al. 2015; Zecchini et al. 2018; Deng et al. 2019). Here, we show that Kazinol C from *Broussonetia kazinoki* stimulates ER stress-associated autophagy, and that co-treatment with Kazinol C and the autophagy inhibitor 3-methyladenine (3-MA) potentiates cancer therapeutic effects by increasing apoptotic cell death.

## Materials and methods

### Reagents

The Kazinol C extraction method and chemical structure have been described previously (Kim et al. 2015). The final concentration of Kazinol C was 15–30 μM to achieve maximal autophagy induction and minimal cytotoxicity. 3-Methyladenine (3-MA; Sigma-Aldrich, USA) and chloroquine (CQ; Sigma-Aldrich, USA) were used to block autophagic flux by impairing autophagosome formation and autophagosome-lysosome fusion, respectively. 4-Phenylbutyric acid (4-PBA; Sigma-Aldrich, USA) was used to block the ER stress signaling pathway.

### Cell culture

Human adult dermal fibroblasts (HADFs; NHDF-Ad-Der Fibroblasts, Lonza, USA), mouse embryonic fibroblasts (MEFs), and the HepG2 hepatocarcinoma cell line were cultured in Dulbecco’s modified Eagle’s medium (DMEM; Gibco, USA) supplemented with 10% fetal bovine serum (FBS; Gibco, USA) and 1% penicillin/streptomycin (Gibco, USA), and maintained at 37°C in a humidified atmosphere with 5% CO_2_. The DU145 prostate cancer cell line was cultured in RPMI 1640 medium (Roswell Park Memorial Institute; Gibco, USA) supplemented with 10% FBS and 1% penicillin/streptomycin. Mouse primary hepatocytes (MPHs) were cultured in DMEM/F-12 (Gibco, USA) supplemented with 10% FBS, 1% penicillin/streptomycin, 100x insulin-transferrin-selenium-sodium pyruvate (Gibco, USA), and 40 ng/ml dexamethasone (Gibco, USA).

### Immunoblot analysis

Proteins separated using SDS-PAGE were transferred onto nitrocellulose membranes (GE Healthcare, USA). The membranes were blocked with 5% nonfat milk in phosphate-buffered saline (PBS) containing 0.1% Tween 20 (PBST; Duchefa, Netherlands). The primary antibodies used for immunoblotting were anti-LC3B, anti-p62, anti-PERK, anti-GRP78/BiP, anti-IRE1alpha, anti-TFEB, anti-ATG5, anti-PARP, and anti-Caspase3 from Cell Signaling Technology (USA). Anti-XBP1 and anti-GAPDH antibodies were purchased from Abcam (UK) and Santa Cruz (USA), respectively. The membranes were then washed with PBST and incubated with peroxidase-conjugated secondary antibodies (Jackson ImmunoResearch, USA). Labeled protein bands were detected and analyzed using an EZ-Western Lumi Pico kit (DoGen, Republic of Korea) and LAS mini-4000 (Fujifilm, Japan).

### Quantitative RT–PCR

Total RNA of cells was extracted using TRIzol (Invitrogen, USA) and reverse transcribed using the RevertAid reverse transcription kit (ThermoFisher, USA) according to the manufacturer’s instructions. The mRNA was amplified using Power SYBR Green PCR Master Mix (Applied Biosystems, USA), and the abundance was detected using the StepOnePlus Real-Time PCR System (Applied Biosystems, USA). The amount of mRNA was calculated using ΔΔCt method and normalized. The primers used are listed in Table 1. Glyceraldehyde-3-phosphate dehydrogenase (GAPDH) was used as an endogenous control.

**Table 1.** Primer sequences for quantitative RT-PCR.

Gene Name	Species	Primer sequence
**CHOP**	Mouse	FW: 5′-CTGCCTTTCACCTTGGAGAC-3′ RV: 5′-CGTTTCCTGGGGATGAGATA-3′
**GRP78/BiP**	Mouse	FW: 5′-CATGGTTCTCACTAAAATGAAAGG-3′ RV: 5′-GCTGGTACAGTAACAACTG-3′
**GADD34**	Mouse	FW: 5′-CCGCTTATCCCACATCAC-3′ RV: 5′-GGTTTGTATCCCGGAGCT-3′
**uXBP1**	Mouse	FW: 5′-AAGAACACGCTTGGGAATGG-3′ RV: 5′-ACTCCCCTTGGCCTCCAC-3′
**sXBP1**	Mouse	FW: 5′-GAGTCCGCAGCAGGTG-3′ RV: 5′-GTGTCAGAGTCCATGGGA-3′
**GAPDH**	Mouse	FW: 5′-TCCCACTCTTCCACCTTCGA-3′ RV: 5′-AGTTGGGATAGGGCCTCTCTTG-3′
**CHOP**	Human	FW: 5′-GCGCATGAAGGAGAAAGAAC-3′ RV: 5′-TCACCATTCGGTCAATCAGA-3′
**GRP78/BiP**	Human	FW: 5′-GCTCGACTCGAATTCCAAAG-3′ RV: 5′-GATCACCAGAGAGCACACCA-3′
**GADD34**	Human	FW: 5′-AAACCAGCAGTTCCCTTCCT-3′ RV: 5′-CTCTTCCTCGGCTTTCTCCT-3′
**uXBP1**	Human	FW: 5′-CCCTCCAGAACATCTCCCCAT-3′ RV: 5′-ACATGACTGGGTCCAAGTTGT-3′
**sXBP1**	Human	FW: 5′-TGCTGAGCTCGCAGCAGGTG-3′ RV: 5′-GCTGGCAGGCTCTGGGGAAG -3′
**GAPDH**	Human	FW: 5′-CTGGGCTACACTGAGCACCAG-3′ RV: 5′-CCAGCGTCAAAGGTGGAG-3′

### Immunofluorescence

MEF and DU145 cells stably expressing mRFP-GFP-LC3 were used to monitor the number of LC3-positive puncta and autophagic flux. Nuclear translocation of TFEB was observed in DU145 cells stably expressing GFP-TFEB. Cells on coverslips were fixed with 4% paraformaldehyde solution (Biosesang, Republic of Korea) for 1 h and washed with PBS. Samples were then mounted with mounting medium containing DAPI (Vector Laboratories, USA), and monitored under a confocal microscope (Zeiss LSM700, Germany). To observe TFEB translocation to the nucleus in HADFs, cells on coverslips were fixed with 4% PFA for 1 h and then washed with PBS. Next, the cells were blocked with 10% FBS in PBS, incubated with TFEB antibody (Cell Signaling Technology, USA) overnight, and then incubated with Alexa Fluor 488 secondary antibody (Invitrogen, USA) for 1 h.

### Measuring apoptosis by flow cytometry analysis

Cells were double-stained using fluorescein isothiocyanate (FITC)-labeled Annexin V and propidium iodide (PI) (Annexin V-FITC Apoptosis Detection Kit I, BD Biosciences, USA) and analyzed by fluorescence-activated cell sorting (FACS). Briefly, cells were washed twice with cold PBS, harvested, and resuspended in 100 μl of 1x Annexin V binding buffer. Cells were then stained with Annexin V-FITC and PI for 5 min in dark, and the extent of apoptosis was measured using a FACSCanto II™ flow cytometer (BD Biosciences, USA). Data were analyzed using the FlowJo software (FlowJo LLC, USA).

### Statistical analysis

All statistical analyses were performed using GraphPad Prism 5 software (GraphPad Software, Inc. USA) All experiments were performed in triplicates. Data are expressed as mean ± S.E.M., and criteria for statistical significance were set at *p* < 0.05 (*p < 0.05, **p < 0.01, ***p < 0.001). Unpaired Student’s t-test and two-way ANOVA were used for the analysis.

## Results

### Kazinol C stimulates autophagic flux

**Figure 1.**
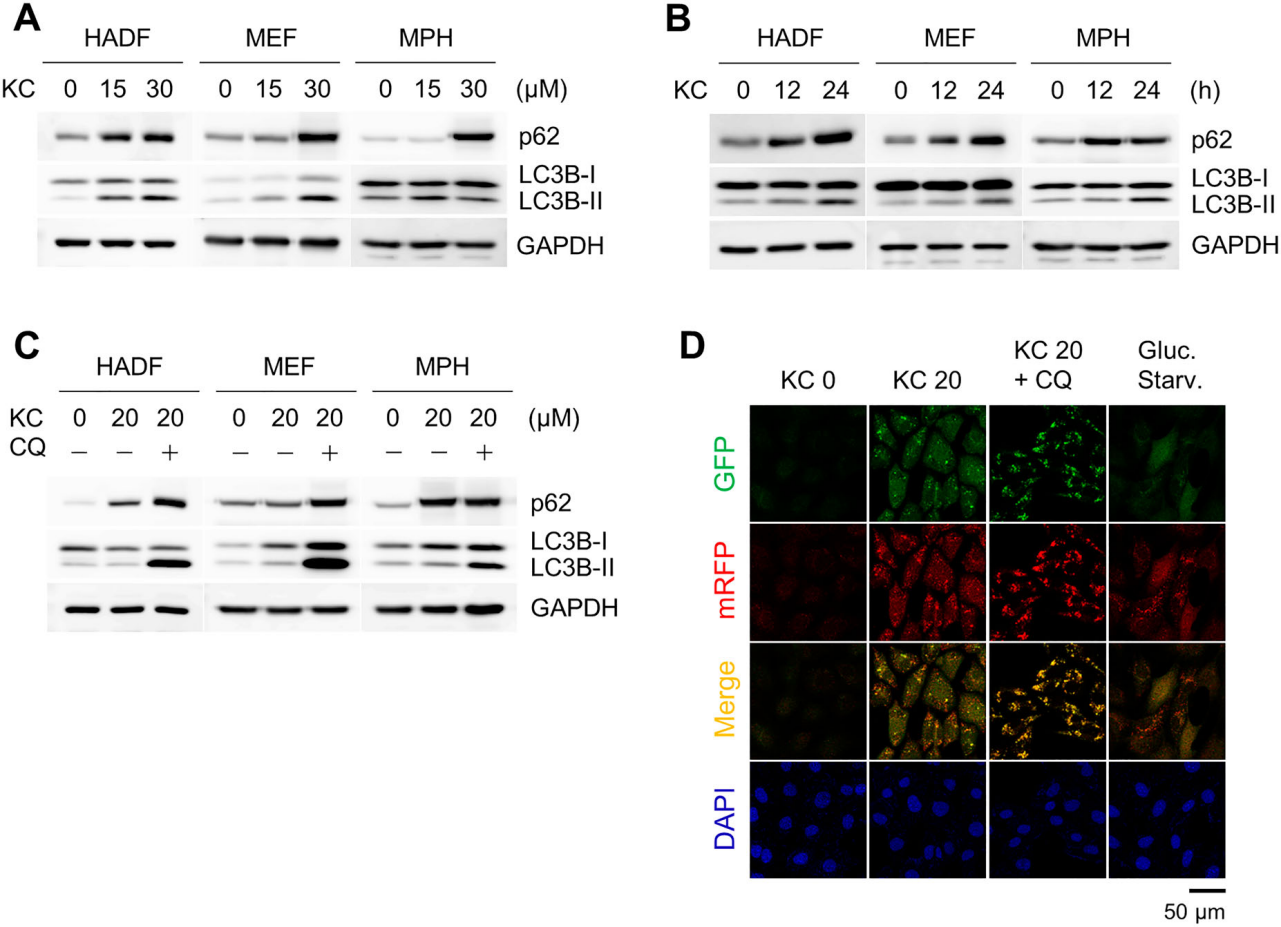
Autophagy induction upon treatment with the natural compound Kazinol C (KC). (A) Autophagy induction by KC (0, 15, or 30 µM) for 24 h in a dose-dependent manner in Human adult dermal fibroblasts (HADFs), mouse embryonic fibroblasts (MEFs) and mouse primary hepatocytes (MPHs). The induction of autophagy is measured by immunoblotting using LC3B antibody. GAPDH is used as a loading control. (B) Autophagy induction by KC (20 µM for 0, 12, or 24 h) in a time-dependent manner. (C) Co-treatment with autophagy inhibitor chloroquine (CQ, 100 µM) and KC (20 µM) blocks autophagic flux. (D) LC3 puncta were observed in mRFP-GFP-LC3 stably expressing MEFs by KC (20 µM for 24 h). Glucose starvation (Gluc. starv., for 6 h) is a positive control. The nuclei were stained with DAPI. Scale bar = 50 µm.

Kazinol C is known to activate AMP-activated protein kinase (AMPK) and induce apoptosis in various cancer cell lines (Kim et al. 2015). AMPK is a major energy sensor in cells, and activation of AMPK is associated with the induction of autophagy (Kim et al. 2011). Therefore, we treated several cell lines with Kazinol C to determine whether the autophagic pathway was activated. First, we treated normal cell lines, including human adult dermal fibroblasts (HADFs), mouse embryonic fibroblasts (MEFs), and mouse primary hepatocytes (MPHs) with Kazinol C. The conversion of LC3-I to II, which occurs by conjugation of LC3-I with phosphatidylethanolamine, is a well-established autophagy marker that measures autophagy flux in the assay (Klionsky, Abdel-Aziz, et al. 2021). LC3 conversion was observed in a time- and dose-dependent manner when challenged with Kazinol C at different times and concentrations (Figure 1(A,B)). Next, the cells were co-treated with chloroquine (CQ), which blocks autophagosome-lysosome fusion and lysosomal degradation. We also detected increased LC3 conversion by Kazinol C and robust LC3B-II accumulation by CQ in various cell types (Figure 1(C)). In addition, the ability of Kazinol C to induce LC3 puncta formation was confirmed in MEFs stably expressing mRFP-GFP-LC3 (Figure 1(D)). These results indicate that Kazinol C induces autophagy in various cell types.

### Autophagy induced by Kazinol C is mediated by ER stress-associated UPR signaling

**Figure 2.**
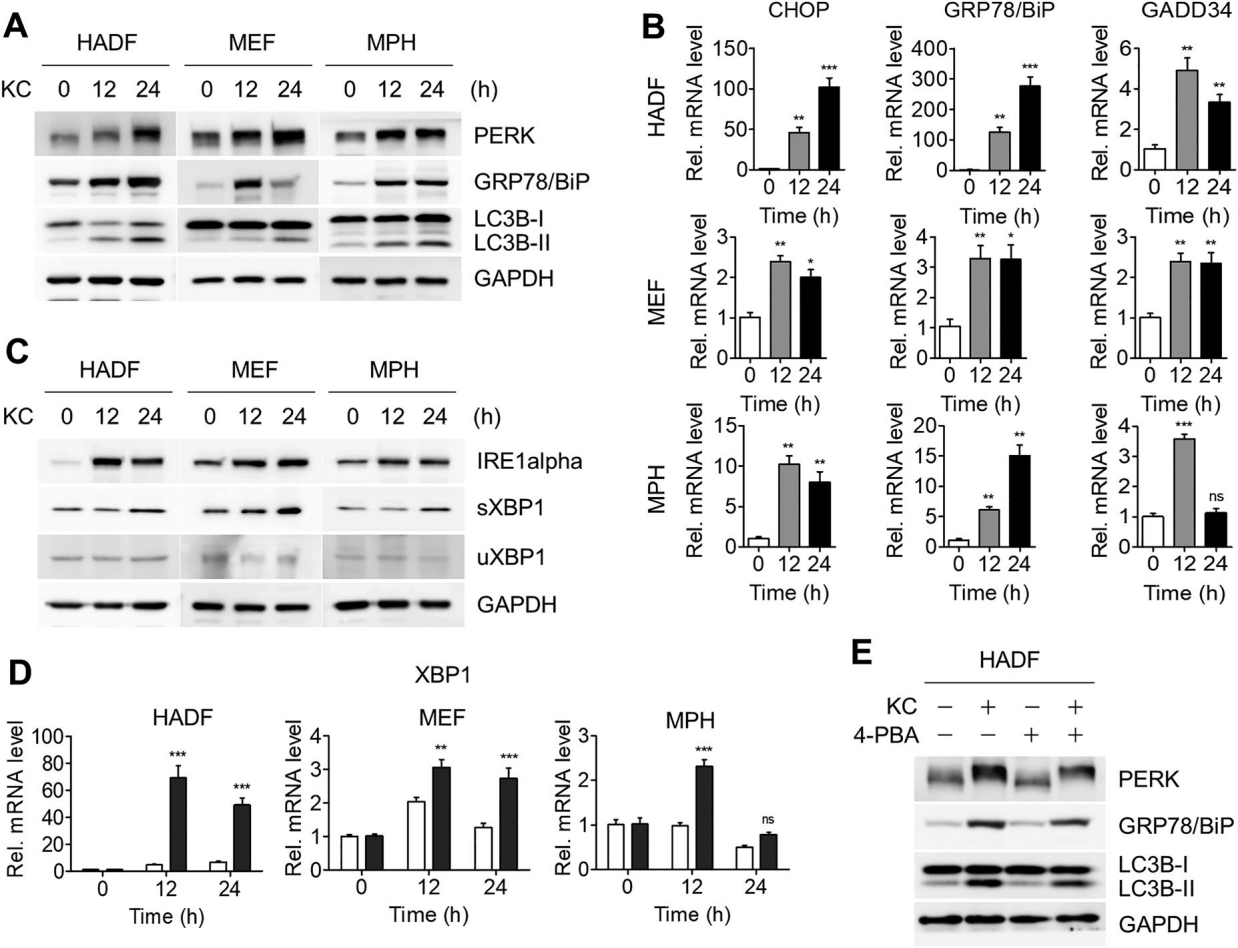
KC-induced autophagy by ER stress-mediated UPR signaling. (A) KC induces ER stress-mediated UPR. The phosphorylation of PERK, the UPR sensor protein, can be observed through the shift of PERK band. GRP78/BiP is the master regulator of UPR. (B) Increased level of IRE1alpha, an ER-localized transmembrane protein and endonuclease, and XBP1 splicing by IRE1alpha were identified. sXBP1, spliced form of XBP1, uXBP1, unspliced form of XBP1. (C) HADF, MEF, and MPH cells were treated with KC for the indicated time (0, 12, or 24 h) and mRNA levels of *uXBP1* and *sXBP1* were determined using qRT-PCR. (D) mRNA levels of *CHOP*, *GRP78/BiP*, and *GADD34* were analyzed by qRT-PCR using the specific primers. All data in bar graphs represent the mean value from three independent experiments. *p < 0.05; **p < 0.01; ***p < 0.001. ns, not significant. (E) HADF cells were treated with either KC (20 µM), 4-PBA (2.5 mM) alone for 16 h, or pre-treated with 4-PBA 4 h before the treatment with KC, and diminished levels of ER stress marker proteins were measured by immunoblotting.

A growing number of studies have been reported on the crosstalk between autophagy and ER stress, and our previous study also showed that another natural compound, DFS, stimulated both the ER stress response and autophagy (Kwon et al. 2020). In addition, the accumulated p62 protein correlated with ER stress (Figure 1(A,B)) (Gonzalez-Rodriguez et al. 2014). To investigate whether Kazinol C triggers both ER stress and autophagy, we treated various cell lines with Kazinol C and examined ER stress-mediated UPR markers, such as protein kinase RNA-like endoplasmic reticulum kinase (PERK), a key UPR signal activator, and GRP78/BiP, a master regulator of the UPR, using immunoblot analysis. The PERK band shifted upon Kazinol C treatment, confirming its autophosphorylation by ER stress; GRP78/BiP expression levels increased in parallel with the time-dependent enhancement in LC3 lipidation (Figure 2(A)). To verify the transcript levels of the UPR-responsive genes, we performed quantitative RT–PCR. Consistent with immunoblot analysis, expression of UPR-responsive genes, including *CHOP*, *GRP78/BiP,* and *GADD34*, was found to have increased upon Kazinol C treatment (Figure 2(B)). In response to UPR, *XBP1* transcript is spliced by the activated endonuclease IRE1α, and the protein encoded by the processed *XBP1* is termed the spliced form of XBP1 (sXBP1), whereas the unprocessed *XBP1* produces the unspliced form of XBP1 (uXBP1) (Calfon et al. 2002). Immunoblot data in Figure 2(C) show increased levels of IRE1α and *XBP1* splicing (increased sXBP1 levels) following Kazinol C treatment in a time-dependent manner. The mRNA levels of the spliced form of *XBP1* were also elevated (Figure 2(D)). Next, using 4-phenylbutyric acid (4-PBA), a selective inhibitor of ER stress, we assessed whether ER stress-mediated UPR was caused by Kazinol C. PERK phosphorylation levels and GRP78/BiP protein levels were reduced upon treatment with 4-PBA and Kazinol C in HADFs (Figure 2(E)). In addition, the conversion of LC3-II by Kazinol C decreased upon treatment with 4-PBA (Figure 2(E)). Taken together, these results suggest that Kazinol C activates ER stress-mediated UPR and that Kazinol C-induced autophagy is mediated, at least in part, by UPR.

### ER stress mediates autophagy via TFEB translocation in cancer cell lines as well as normal cell lines

**Figure 3.**
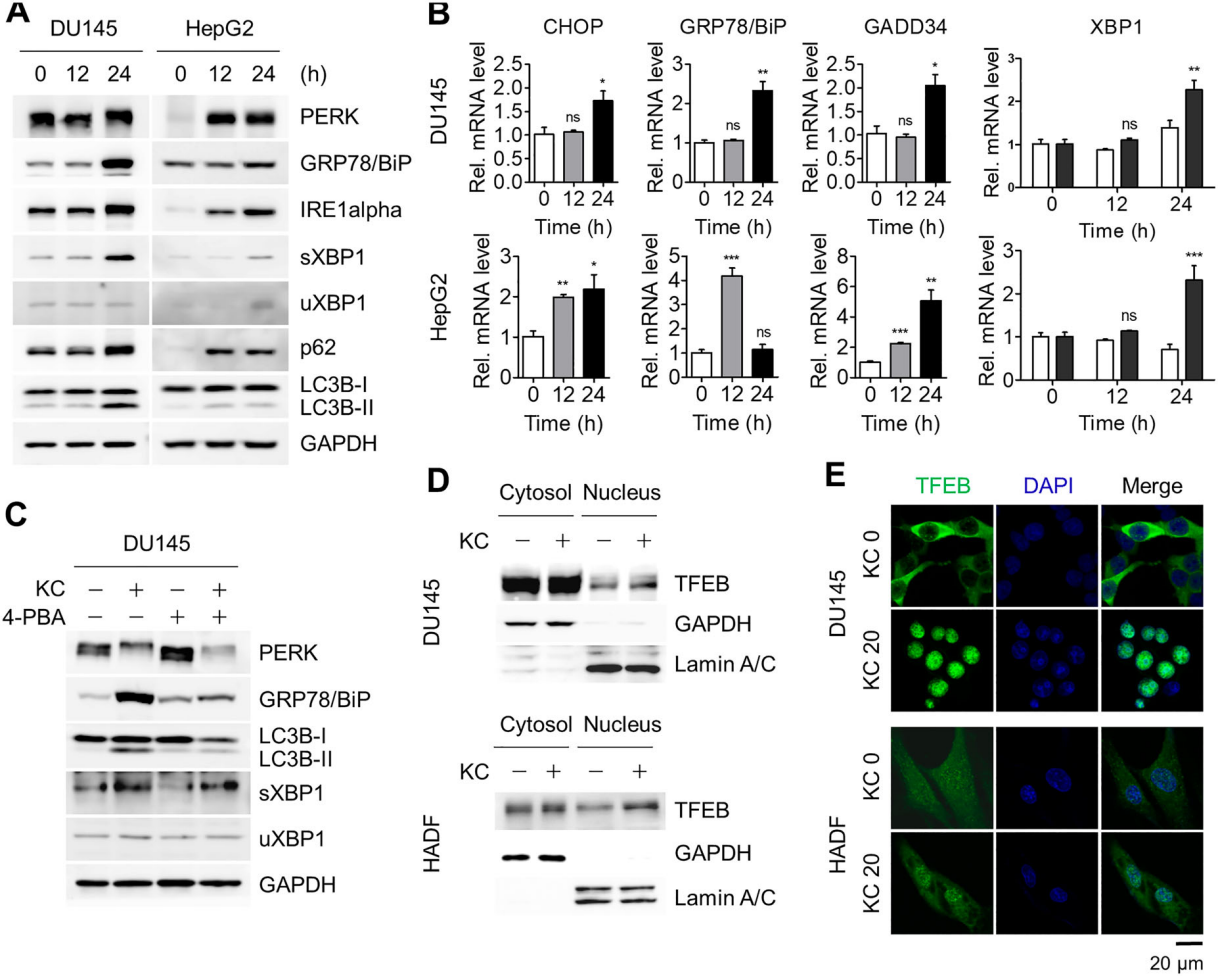
Induction of both autophagy and UPR in cancer cell lines. (A) DU145 and HepG2 cells were treated with KC (20 µM for 0, 12, or 24 h) and ER stress-associated UPR signaling proteins were detected. (B) mRNA levels of *CHOP*, *GRP78/BiP*, *GADD34*, *sXBP1*, and *uXBP1* were measured by qRT-PCR. (C) DU145 cells were treated with either KC (20 µM), 4-PBA (5 mM) alone for 16 h, or pre-treated with 4-PBA 4 h before the treatment with KC, and diminished levels of ER stress marker proteins were analyzed by immunoblotting. (D) DU145 cells stably overexpressed by TFEB were treated with KC (20 µM) for 24 h. HADF cells were treated with KC and TFEB translocation (endo) from cytosol to nucleus was determined through subcellular fractionation followed by immunoblotting. (E) The cells were treated with KC and immunostained with anti-TFEB and anti-rabbit (H + L) secondary antibody conjugated with FITC (Green). Cell nuclei were stained with DAPI (Blue). Scale bar = 20 µm.

To investigate whether ER stress-mediated autophagy by Kazinol C is also induced in cancer cell lines, DU145 prostate cancer and HepG2 hepatocellular carcinoma cell lines were treated with Kazinol C. Consequently, Kazinol C treatment increased UPR signaling based on PERK phosphorylation, elevated protein levels of GRP78/BiP, IRE1α, and sXBP1, and enhanced target gene expression of UPR signaling, including *sXBP1* (Figure 3(A,B)). LC3-II conversion also increased in Kazinol C-treated cancer cells (Figure 3(A)). As in normal cells, 4-PBA reduced the extent of Kazinol C-induced UPR signaling as well as LC3-II conversion in cancer cells (Figure 3(C)).

To further explore the autophagy pathway, we monitored the nuclear localization of TFEB, a master regulator of gene expression involved in autophagy and lysosomal biogenesis, in DU145 cells stably expressing GFP-TFEB as well as in HADFs. TFEB appears to have regulatory functions in the autophagy pathway, particularly by controlling genes belonging to the coordinated lysosome expression and regulation (CLEAR) network (Settembre and Ballabio 2011; Settembre et al. 2011). Kazinol C treatment increased the amount of nuclear TFEB (Figure 3(D)). The nuclear translocation of TFEB by Kazinol C treatment was more clearly detected by immunofluorescence assays (Figure 3(E)). The majority of TFEB was localized in the cytoplasm without Kazinol C treatment, whereas it was translocated to the nucleus upon Kazinol C treatment in both cell types (Figure 3(E)). Taken together, these results indicate that Kazinol C induces ER stress-associated UPR and TFEB activation in both normal and cancer cells.

### Inhibition of autophagic flux can lead to apoptotic cell death

**Figure 4.**
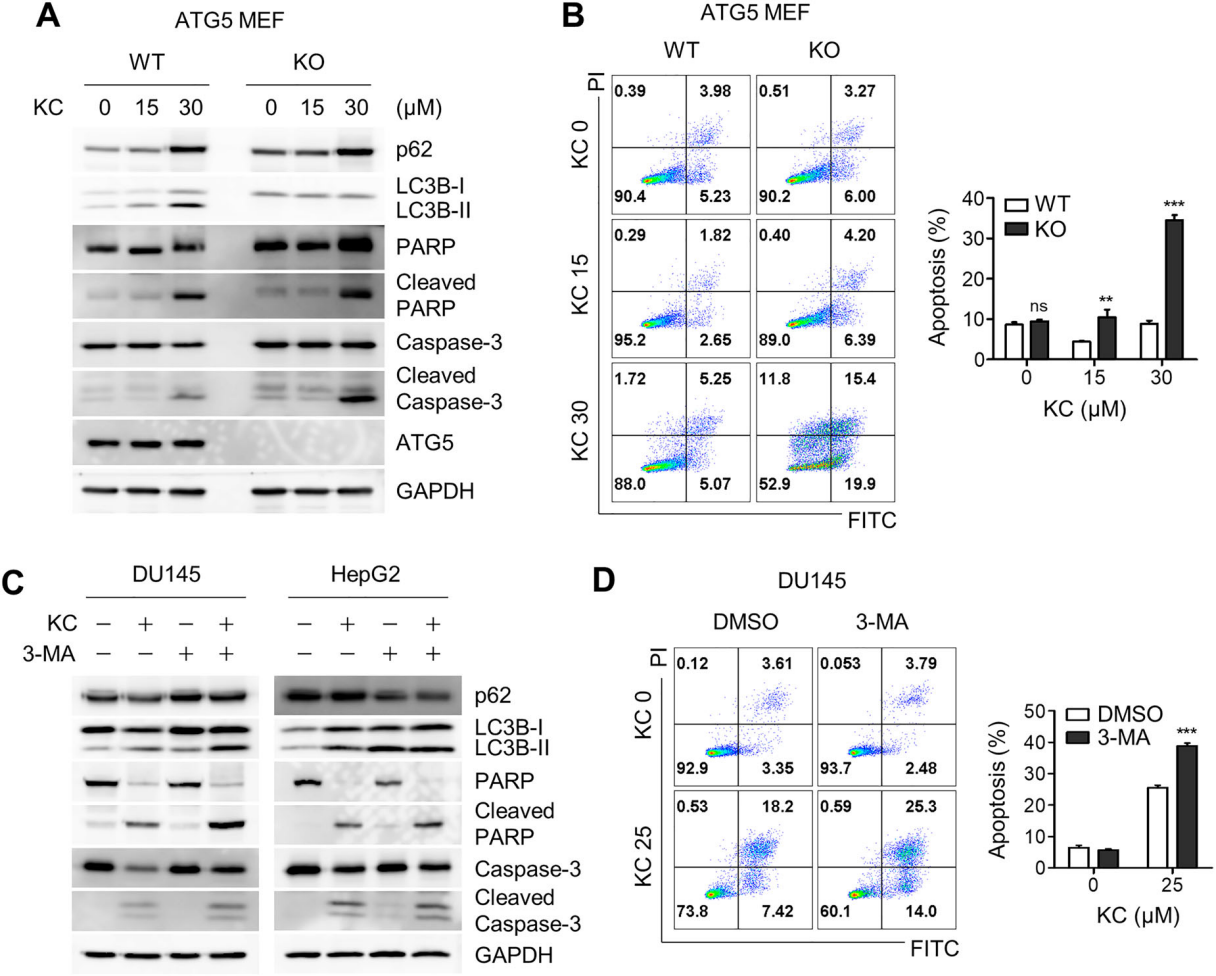
Increased apoptotic cell death by the blockage of autophagic flux. (A) ATG WT and KO MEF cells were treated with KC (15 or 30 µM) for 24 h, and PARP and Caspase-3 cleavage were measured through immunoblotting using the specific antibodies. (B) Cell death in ATG5 WT and KO cells were analyzed after the treatment with KC by FACS. (C) DU145 and HepG2 cells were treated with either KC (30 µM), 3-Methyladenine (3-MA, 5 mM) alone, or pre-treated with 3-MA (5 mM) 10 h before the treatment with KC (30 µM), and apoptotic cell death marker proteins were detected by immunoblotting. (D) DU145 cells were treated either KC (25 µM), 3-Methyladenine (3-MA, 5 mM) alone, or pre-treated with 3-MA (5 mM) 10 h before the treatment with KC (25 µM), and cell death was measured after 16 h of the treatment using FACS analysis. All data in bar graphs throughout the figures represent the mean value obtained from three independent experiments. *p < 0.05; **p < 0.01; ***p < 0.001. ns, not significant.

ATG5 plays a critical role in the early stages of autophagic flux, particularly in the formation of autophagosome precursors by conjugating with ATG12 and ATG16 (Longatti et al. 2010; Moreau et al. 2011). Thus, autophagy is significantly inhibited in ATG5 knockout cells. To investigate whether autophagy inhibition induces apoptotic cell death, we treated ATG5 wild-type and knockout (KO) MEFs with Kazinol C. As expected, autophagic flux was deficient in ATG5 KO cells compared to WT MEFs, and apoptosis markers such as cleaved caspase-3 and PARP were significantly enhanced in ATG5-deficient cells (Figure 4(A)). To further investigate this observation, flow cytometry-based apoptosis analysis was performed using Annexin V and propidium iodide (PI) apoptosis detection kit. Consistent with the results obtained during immunoblotting analysis, considerably more apoptotic cell death occurred in KO cells, mostly at the early stages of apoptosis (Figure 4(B)). To test whether autophagy inhibition triggers apoptotic cell death in cancer cells, we used the autophagy inhibitor 3-MA to block autophagosome formation in DU145 and HepG2 cancer cell lines. Immunoblot analysis revealed enhanced cleavage of caspase-3 and PARP (Figure 4(C)), similar to that observed in ATG5-deficient cells. In addition, an increase in apoptotic cell death was observed when cells were treated with Kazinol C along with 3-MA (Figure 4(D)). In conclusion, blockade of autophagic flux with 3-MA potentiates Kazinol C-induced ER stress-associated apoptotic cell death, although its effect was weaker in cancer cells.

## Discussion

The focus of our research is on the induction of autophagy by natural compounds and their synergistic effects on cancer; and in the present study, we identified Kazinol C as a novel inducer of autophagy. Previously, we have found that DFS, a plant-derived compound, activated UPR signaling and autophagy with concomitant translocation of TFEB to the nucleus (Kwon et al. 2020). The mode of action of Kazinol C is very similar to that of DFS, in that it activates the UPR signal, induces autophagy, and promotes the translocation of TFEB to the nucleus; however, the underlying molecular details remain unknown. In addition, both compounds can induce apoptotic cell death when autophagy is inhibited, otherwise the cells would not die. In ATG5 knockout MEFs in which autophagic flux is completely blocked, both Kazinol C and DFS induced significant levels of apoptosis, confirming a pro-survival role of autophagy in stress-induced response situations. Chemical inhibition of autophagy in cancer cells also resulted in an increase in cell death upon treatment with Kazinol C or DFS, but the efficacy of cell death was much better with DFS treatment. It will be interesting to investigate in details the proteins that Kazinol C and DFS target to induce these cellular events. ER stress inducers, such as tunicamycin and arachidonic acid, induce apoptosis, as Kazinol C does in cancer cells (Bae et al. 2020; Kong et al. 2020). Therefore, blocking autophagy along with the induction of ER stress would be a more effective way to induce cancer cell death.

AMPK is a master regulator of energy metabolic pathways and maintains cellular homeostasis under various stress conditions (Kim et al. 2016). AMPK also plays a central role in the regulation of autophagy. For example, AMPK directly modulates ULK1 (Unc-51 like autophagy activating kinase) and PI3 K (phosphatidylinositol 3 kinase) at the autophagy initiation stage (Kim et al. 2011). Recent studies have shown that the AMPK-SKP2-CARM1 signaling axis is involved in the induction of autophagy following glucose starvation (Shin et al. 2016). Kazinol C is known to induce apoptosis in HT-29 colorectal cancer cells by activating AMPK, and AMPK inhibition by Compound C is known to block apoptosis caused by Kazinol C (Kim et al. 2015). We attempted to determine whether AMPK activity was required for the induction of autophagy by Kazinol C using Compound C as an AMPK inhibitor, but found that autophagy was induced when Compound C alone was used for the treatment (data not shown). After reviewing the literature, it was found that Compound C induces autophagy independently of AMPK inhibition in cancer cells (Vucicevic et al. 2011). Therefore, the effect of Compound C in preventing apoptosis may be achieved through the inhibition of AMPK and induction of autophagy, which plays a pro-survival role.

Polyphenols such as Kazinol C may also be a good therapeutic strategy for diabetes and metabolic syndrome. For instance, resveratrol, curcumin and berberine are known to modulate mitochondrial functions (Kim et al. 2016). Impairment of autophagy also promotes dysregulation of insulin signaling, leading to impaired glucose clearance and ultimately metabolic syndrome-associated insulin resistance (Jahng et al. 2019). Kazinol C has been implicated in the prevention of cytokines and type I diabetes-induced pancreatic β cell injury (Bae et al. 2015). Therefore, Kazinol C may be an effective therapeutic intervention for both cancer as well as metabolic diseases.
